# Metagenome-assembled genomes of the methanogenic enrichment obtained from drilling fluid wastes stored in permafrost

**DOI:** 10.1128/mra.01159-23

**Published:** 2024-04-11

**Authors:** V. A. Shcherbakova, V. I. Rechkina, V. E. Trubitsyn

**Affiliations:** 1 Skryabin Institute of Biochemistry and Physiology of Microorganisms, Pushchino Scientific Center for Biological Research of the Russian Academy of Sciences, Pushchino, Moscow, Russia; Montana State University, Bozeman, Montana, USA

**Keywords:** permafrost, metagenome-assembled genomes, methanogens

## Abstract

We studied methanogenic enrichment (Kha) from spent drilling fluid stored in permafrost, Kharasavey (71°10′50″N 66°51′50″E) gas field located in the western part of the Yamal Peninsula. The metagenome-assembled genomes showed that Kha consists of representatives of *Methanosarcina*, *Methanobacterium*, *Proteinifillum*, and *Synergistetes* genera.

## ANNOUNCEMENT

The drilling fluid (DF) (70 g) sample was collected using a sterile sampler into a sterile container and transported to the laboratory at –20°C. After a half-year anaerobic incubation in a 150-mL glass flask at room temperature, 1 mL of fermented DF was transferred in 20 mL of DSMZ 141 medium (1) with CO_2_:H_2_ (80:20) and methanol as substrates and was anaerobically incubated at 20°C for a month. After four transfers in the same conditions, we received the stable methanogenic enrichment Kha.

Genomic DNA preparation and sequencing were performed by BioSpark Company (Troitsk, Russia). Genomic DNA from Kha was isolated with the FastDNA spin kit (MP Biomedicals, USA) using the column method and deposition on silica gel. The paired-end libraries were synthesized using KAPA HyperPlus kits (Kapa Biosystems, USA) in accordance with the manufacturer’s recommendations. Sequencing was performed on the Illumina NovaSeq 6000 platform, and a paired-end library with a total of 17,702,025 reads and a read length of 150 bp with a mean quality of 35 was obtained.

To obtain metagenome-assembled genomes (MAGs), the Kbase online platform with appropriate tools was used (2). The quality of the reads was assessed using the FastQC v.0.12.1 program (3). Reads were processed in Trimmomatic (4) with the parameters “HEADCROP = 15, MINLEN = 50” and removing adapters from the TruSeq3-PE-2 set. Contigs and scaffolds were assembled from paired libraries using MetaSPAdes v.3.15.3 with default parameters (5). Contig binning was performed using the CONCOCT v.1.1 (6) with subsequent optimization of the result in DAS Tool v.1.1.2 with BLAST gene identification (7). The completeness of the obtained genomes was assessed using CheckM v.1.0.18 (8), and taxonomic affiliation was determined in GTDB-tk v.2.3.2 (9). The orthoANI calculation was made using the online service https://www.ezbiocloud.net/tools/ani. Alignment of reads to genomes and determination of coverage and the number of aligned reads were carried out using bowtie2 v. 2.3.2 (10). Default parameters were used for all software unless otherwise noted. Rapid annotation of genomes was carried out with the CBI Prokaryotic Genome Annotation Pipeline (11).

As a result of the work, four MAGs were obtained, two of which belonged to methanogenic archaea (*Methanosarcina* and *Metanobacterium* genera), and two belonged to *Bacteria* (Table 1).

**TABLE 1:** Description of MAGs obtained during the study

Genome feature	MAGs			
	IBPM_KMB_001	IBPM_KMB_002	VO1	IBPM_KMB_004
Closest genome (GTDBtk data)	GW-*Synergistetes*-1, MAG GCA_002839185.1	*Proteiniphilum saccharofermentans* M3/6^T^ GCF_900095135.1	*Methanosarcina* sp. 2.H.T.1A.6, MAG GCF_000979455.1	*Methanobacterium* sp. Maddingley MBC34, MAG GCA_000309865.1
OrthoANI with closest genome (%)	91.06	95.34	84.56	86.26
Completeness/contamination (CheckM)	100/0	99.45/0	99.84/0.65	98.93/0.8
Genome coverage	166 ± 33	603 ± 224	383 ± 146	40 ± 11
Read abundance (%)	2,457,644 (6.94)	15,325,445 (43.29)	11,055,016 (31.23)	679,997 (1.92)
Genome length (bp)	2,214,899	3,800,659	4,322,233	2,538,249
Scaffolds	17	59	144	9
*N*_50_	396,354	102,999	44,010	489,195
*L*_50_	3	13	30	3
GC content (%)	46.8	43.8	41.6	39.2
Genes (total)	2,065	3,039	3,630	2,505
CDS^*a*^ (total)	2,016	2,993	3,571	2,453
Genes (coding)	2,009	2,957	3,518	2,448
tRNA	45	44	57	46
16S, 5S, 23S rRNA	0, 1, 0	–	–	2, 1, 1

^*a*^ Protein coding sequence (CDS).

Of the resulting assemblies, one (IBPM_KMB_004) was a high-quality MAG (completeness was more than 90%; contamination was less than 5%; 16S, 5S, 23S, and at least 16 tRNA were present). The remaining three drafts were close to high-quality MAGs, except for the absence of 16S/5S/23S rRNA in three representatives (12). All MAGs demonstrated a large difference in the orthoANI coefficient with the closest relatives and may belong to new bacterial and archaeal taxa.

## Data Availability

Raw sequence reads are available in the National Center for Biotechnology Information (NCBI) GenBank database under Sequence Read Archive accession numbers SRR26631526 (forward library) and SRR26631527 (reverse library) (BioProject PRJNA1034487 and BioSample SAMN38056839). The obtained draft genomes were deposited in the NCBI under the WGS master record numbers JAXQGB000000000.1 (IBPM_KMB_001), JAXQGC000000000.1 (IBPM_KMB_002), JAXQGD000000000.1 (VO1), and JAXQGE000000000.1 (IBPM_KMB_004). Processing reads and obtaining draft genomes were recreated in the Kbase public narrative IBPM_KMB_Methagenome (https://narrative.kbase.us/narrative/171292).
